# Network pharmacology suggests biochemical rationale for treating COVID-19 symptoms with a Traditional Chinese Medicine

**DOI:** 10.1038/s42003-020-01190-y

**Published:** 2020-08-18

**Authors:** Deng-hai Zhang, Xue Zhang, Bin Peng, Sheng-qiong Deng, Yu-fang Wang, Lin Yang, Kai-zheng Zhang, Chang-quan Ling, Kun-lun Wu

**Affiliations:** 1 Shanghai Health Commission Key Lab of Artificial Intelligence (AI)-Based Management of Inflammation and Chronic Diseases, Sino-French Cooperative Central Lab, Shanghai Pudong Gongli Hospital, Secondary Military Medical University, 200135 Shanghai, China; 2 Post-graduate Training Base in Shanghai Gongli, Post-Graduate College, Ningxia Medical University, 750004 Yinchuan, Ningxia Province China; 3 Department of Research Affair Management, Shanghai Pudong Gongli Hospital, Secondary Military Medical University, 200135 Shanghai, China; 4 The State Key Laboratory of Bioreactor Engineering, New World Institute of Biotechnology, East China University of Science and Technology, 200237 Shanghai, China; 5 The Traditional Chinese Medicines of Changhai Hospital, Secondary Military Medical University, 200433 Shanghai, China; 6 Department of Traditional Chinese Medicine, Shanghai Pudong Gongli Hospital, China, Secondary Military Medical University, 200135 Shanghai, China

**Keywords:** Pharmacology, Computational biology and bioinformatics, Viral infection, SARS-CoV-2

## Abstract

Chinese herbal formulas including the lung-cleaning and toxicity-excluding (LCTE) soup have played an important role in treating the ongoing COVID-19 pandemic (caused by SARS-CoV-2) in China. Applying LCTE outside of China may prove challenging due to the unfamiliar rationale behind its application in terms of Traditional Chinese Medicine. To overcome this barrier, a biochemical understanding of the clinical effects of LCTE is needed. Here, we explore the chemical compounds present in the reported LCTE ingredients and the proteins targeted by these compounds via a network pharmacology analysis. Our results indicate that LCTE contains compounds with the potential to directly inhibit SARS-CoV-2 and inflammation, and that the compound targets proteins highly related to COVID-19’s main symptoms. We predict the general effect of LCTE is to affect the pathways involved in viral and other microbial infections, inflammation/cytokine response, and lung diseases. Our work provides a biochemical basis for using LCTE to treat COVID-19 and its main symptoms.

## Abstract (web summary)

Deng-hai Zhang et al. report a network pharmacology analysis of the Traditional Chinese Medicine (TCM) lung-cleaning and toxicity-excluding (LCTE) soup. By exploring the chemical compounds that make up LCTE and the likely pathways and proteins that they target, the authors show that LCTE may have the potential to directly inhibit the SARS-CoV-2 virus and relieve inflammatory symptoms.

## Introduction

Towards the end of December 2019, a new type of pneumonia (COVID-19), first reported in Wuhan, China, was identified. It is caused by the novel coronavirus SARS-CoV-2 and transmitted from human-to-human^1–3^. This virus has impacted countries worldwide within a very short time, prompting the World Health Organization (WHO) to pronounce it a worldwide pandemic on March 14, 2020. According to the WHO Daily Report, there have been a total of 142,649 confirmed COVID-19 cases with 5393 deaths in nearly 135 countries, areas or territories as of this writing on March 14, 2020. This pandemic is ongoing, so quickly identifying new preventive and therapeutic agents is a top priority.

Specific vaccines and antiviral agents are the most effective methods for preventing and treating viral infections, yet no such anti-SARS-CoV-2 agents are currently available. Development of these treatments may require months or years, meaning that a more immediate treatment should be found if at all possible. Herbs used in Traditional Chinese Medicine (TCM) present a potentially valuable resource to this end. The effectiveness of herbal treatment in controlling contagious disease was demonstrated during the 2003 severe acute respiratory syndrome (SARS) outbreak^4^. As such, the Chinese government has encouraged the use of herbal medication in fighting this new viral pneumonia, which has brought about good clinical results^5^. The State Administration of TCM reported to official media (chinadaily.com.cn, March 6, 2020) that up to February 17 a total of 60,107 patients with SARS-CoV-2 infections, accounting for 85.2% of the infections, had been treated with TCM nationwide. The Chinese government, encouraged by the evident clinical benefits^5^, has recommended several herbal formulas in its continuously modified plans to prevent and treat SARS-CoV-2 infection. Among these the lung-cleaning and toxicity-excluding soup formula (LCTE, called *Qing Fei Pai Du Tang* in China) has the highest recommendation, based on its clinical effectiveness^6^ and simple preparation (the preparation protocol is provided as Supplementary Data 1). Containing 20 herbal plants and one mineral component (detailed in the following section), LCTE has been officially recommended by Chinese government since February 6, 2020 and remains on the list of recommended treatments in the 7th, most recent version of the National Plan for Preventing and Treating SARS-CoV-2 Infection.

At present, the occurrence rate for COVID-19 infection is declining in China while soaring in some other parts of the world. The successful application of LCTE in China suggests this formula as an attractive means for fighting COVID-19. Yet, the rationale for LCTE application may be difficult to understand when explained in terms of TCM, making it less approachable to modern medical society, especially outside of China. This barrier severely limits the potential benefits gained by introducing LCTE treatment to medical professionals treating COVID-19 worldwide.

Fortunately, thanks to advancements in identifying the chemical compounds contained in Chinese herbs^7–9^ and the emergence of network pharmacology technology^10^, the mechanisms behind LCTE’s effects may be explained through chemical biology. It is well realized that the therapeutic effects of herbal treatments are due to the pharmacological compounds contained in them^11^. For example, it has been proven that the anti-malarial effect of *Artemisia apiacea* is due to its component artemisinin^12^. Likewise, the curative effects of LCTE likely reside in the chemical compounds contained in the formula’s plant ingredients. Network pharmacology highlights a multiple drug component/multiple target model over the single component/single target model in drug development and assessment^10^. The use of this modeling has evolved and expanded over the last several decades^13^. Traditional Chinese herbal formulas, which potentially contain many active compounds, are a typical example of the network pharmacology paradigm^14,15^.

In this work, we investigate the potential working mechanisms behind LCTE’s effectiveness in modern biochemical language. We achieved this by screening the compounds of related plants and undertaking a network pharmacology analysis. Our work shows that two of the main compounds contained in LCTE have the potential to directly inhibit SARS-CoV-2, and that most of the active constituent compounds are anti-inflammatory. Moreover, these compounds evidently target the proteins related to the main symptoms of COVID-19. The general in vivo therapeutic effect of LCTE is predicted to be regulation of pathways related to viral and other microbial infection, inflammation/cytokine response, and lung diseases.

## Results

### Main chemical compounds and protein targets of LCTE

It is well held that Chinese herbal plants contain bioactive compounds and that the therapeutic effects of herbal treatments are achieved via compound/target interaction. We started our work by researching the chemical compounds contained in LCTE and their protein targets. The LCTE formula consists of 20 herbal ingredients and one mineral material (raw gypsum, Rudis Gypsi Miscueris; Table 1). The main component of raw gypsum is inorganic CaSO_4_·2H_2_O, and the inclusion of gypsum in the formula is understandable considering findings that it reduces body temperature^16^ and attenuates heat-induced hypothalamic inflammation via down-regulation of IL-1β^17^. As such, we focused our study on the 20 herbal plants used to formulate LCTE. We found a complete chemical compound list for each plant as recorded in three Chinese herbal databases^7–9^ (detailed in the Methods section). The listed compounds were then filtered using ADME (absorption, distribution, metabolism, and excretion) indices to find which might be absorbable via oral administration^18^. ADME filtration for oral bioavailability is necessary in that most TCM formulations are prepared by boiled herbs with water and the resultant soup is then orally administered. The passing rates ranged from 0.026 to 0.37 with an average of 0.11 (Table 1), indicating that the majority of the compounds present in the related plants would not be absorbed. After finding which compounds were most likely to be absorbed, we again checked the three Chinese herbal databases to find which proteins are affected by each of these compounds. Each herbal ingredient, their total number of compounds, number of orally absorbable compounds, and their number of protein targets are listed in Table 1. More detailed information on the plant contained compounds passing filtration and the proteins targeted by compounds are available in Supplementary Data 2 and Supplementary Data 3.

**Table 1:** The constitution and protein targets of LCTE.

Chinese names	Latin names	Dosage (g)	No. of total ingredients	No. of ingredients passing ADME filtration	No. of protein targets
*Bai Zhu*	Rhizoma Atractylodis Macrocephalae	9	117	7	35
*Ban Xia*	Rhizoma Pinelliae	9	138	12	206
*Chai Hu*	Radix Bupleuri	16	356	11	192
*Chen Pi*	Pericarpium Citri Reticulatae	6	79	6	72
*Dong Hua*	Farfarae Flos	9	160	16	185
*Fu Ling*	Poria	15	89	7	29
*Gan Cao*	Radix Glycyrrhizae	6	283	73	231
*Gui Zhi*	Cinnamomi Ramulus	9	245	7	90
*Huang Qin*	Radix Scutellariae	6	155	33	116
*Huo Xiang*	Pogostemon Cablin	9	122	12	180
*Ma Huang*	Herba Ephedrae	9	373	17	227
*Shan Yao*	Rhizoma Dioscoreae	12	120	13	70
*She Gan*	Rhizoma Belamcandae	9	108	11	104
*Sheng Jiang*	Rhizoma Zingiberis Recens	9	191	5	50
*Xi Xin*	Herba Asari	6	157	9	108
*Xing Ren*	Semen Armeniacae Amarum	9	42	15	113
*Ze Xie*	Rhizoma Alismatis	9	61	7	6
*Zhi Shi*	Fructus Aurantii Immaturus	6	91	14	123
*Zhu Ling*	Polyporus Umbellatus	9	31	8	5
*Zi Wan*	Radix Asteria	9	305	10	206
*Sheng Shi Gao*	Rudis Gypsi Miscueris	15–30	/	/	/

### Antiviral and anti-inflammatory potential of main compounds

**Fig. 1:**
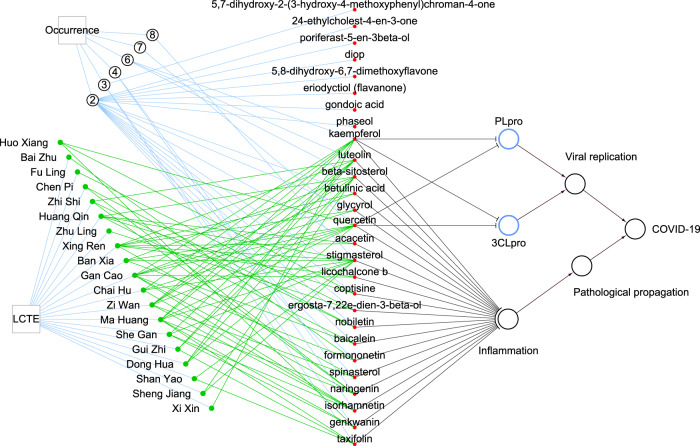
The virus-inhibiting potential and anti-inflammatory property of the main chemical components in LCTE. The circled number indicates the number of occurrences of compounds contained in multiple plants, for example, quercetin is contained in seven plants. Black lines with a blunt end indicate that the source vertex has inhibiting effects on the target vertex, while those ending in an arrow have activation effects. Green networks connect herbal ingredients to the main chemical compounds contained in the plants. PLpro and 3 CLpro stand for papain-like protease and 3C-like protease of SARS-CoV-2, respectively.

In all, the 20 herbal ingredients in LCTE contain 207 different chemical components absorbable through oral administration. In total, 27 of these components are present in at least 2 of the plant sources. Five components were of high concurrence, existing in 6 or more plant sources (Supplementary Data 4). Stigmasterol was present in eight plants, quercetin was present in seven plants, and luteolin, beta-sitosterol, and kaempferol were present in six plants (Fig. 1). Also worth noting is that we recently found two of the five most prevalent compounds, kaempferol and quercetin, have the potential to directly inhibit papain-like protease (PLpro) and 3C-like protease (3CLpro), two enzymes critical to the replication of the COVID-19-causing pathogen SARS-CoV-2^19^.

COVID-19’s basic pathology is viral-caused inflammation. Based on publications in PubMed, we found that 19 of the 27 compounds present in 2 or more of the LCTE herbal plants possess anti-inflammatory properties (Supplementary Data 4). The indication is that the main compounds of LCTE have the potential to directly inhibit SARS-CoV-2 and down-regulate inflammation (Fig. 1).

### Protein enrichment for COVID-19 related symptoms

Fifteen main symptoms have been reported in COVID-19 patients^20,21^. These include fever, cough, myalgia, fatigue, dyspnea, among others, and may be catalogued as general, respiratory and digestive symptoms. The LCTE formula’s clinical effectiveness in relieving these symptoms has been reported in China^6^. To understand the molecular basis for LCTE’s relief of COVID-19 symptoms, we used DisGeNET^22^ to download the proteins related to each symptom and then studied the correspondence between these and the protein targets of LCTE’s orally-absorbable compounds. The exact proteins related to each symptom and the results for the DisGeNet search query ‘viral respiratory infection’ are available in Supplementary Data 5.

**Fig. 2:**
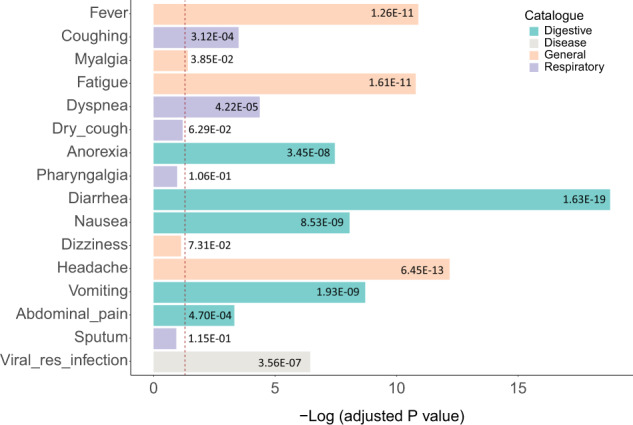
Enrichment of compound-targeted proteins and proteins related to symptoms. The enrichment is represented by the probability (*P*) value of hypergeometric distribution for overlap between chemically-targeted proteins and proteins related to symptoms or proteins related to viral respiratory infection. The bar colors correspond to the anatomic system affected by each symptom. The vertical dotted line indicates the threshold (*P* = 0.05) and the numbers in each bar are the exact adjusted *P* values from enrichment analysis.

Hypergeometric distribution probability computation showed that the component-targeted proteins were significantly enriched in 11 of the symptoms-related proteins (adjusted *P* < 0.05) (Fig. 2; Supplementary Data 6), including 5 of the highest occurrence symptoms: fever, cough, myalgia, fatigue, and dyspnea. The protein sets for anoxia, diarrhea, nausea, headache, vomiting, and abdominal pain were also highly correlated to proteins targeted by LCTE. Computational enrichment for dry cough, pharyngalgia, dizziness, and sputum proteins did not yield significant results, which may be due to the low number of proteins recorded for these symptoms in the DisGeNET database. The proteins related to the search query ‘viral respiratory infection’ in DisGeNET also had high correlation with LCTE-targeted proteins. Since COVID-19 is a viral respiratory disease, such results further support the effectiveness of LCTE in treating COVID-19.

### Key compounds and proteins for LCTE’s symptoms relief

**Fig. 3:**
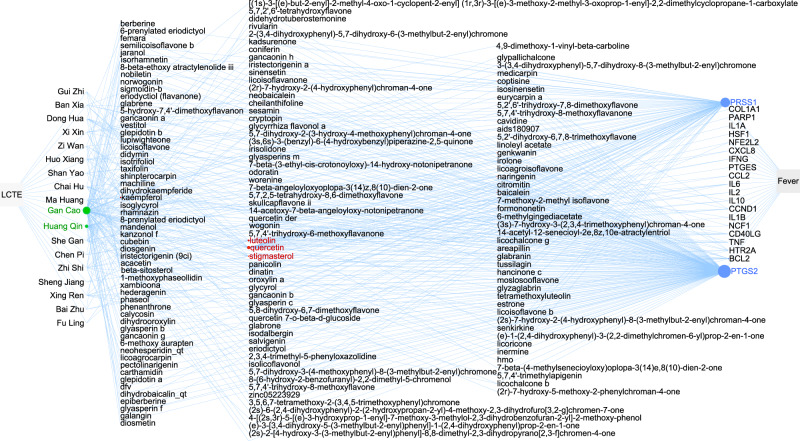
The network of LCTE herbal ingredients, chemical compounds, and target proteins for fever relief. Vertex sizes are proportional to their degree. Vertices with higher degrees indicate a more important role in the network. Green, red and blue vertices represent plants, chemical compounds, and protein targets, respectively. PPSS1 stands for prostaglandin-endoperoxide synthase and PRSS1 for serine protease 1.

The field of network pharmacology suggests that the effects of a drug containing multiple components can be predicted by a network constructed of the linkages between the multiple components and their targets^10^. Inspired by this methodology, we built a network using the following linkages: herbal plants and their corresponding chemical compounds, chemical compounds and their affected proteins, and proteins related to each symptom. By calculating the degree for each vertex, we were able to derive which plants, compounds, and proteins are key to LCTE’s symptom alleviation. For example, in LCTE there are two herbal ingredients, Gan Cao and Huang Qin, and 3 compounds (quercetin, luetolin, stigmasterol) useful in treating fever, and the key proteins affected by these components are PTGS2 (prostaglandin-endoperoxide synthase 2) and PRSS1 (serine protease 1) (Fig. 3). While for treating coughing, two of LCTE’s component plants (Gan Cao, Huang Qin) and five compounds (quercetin, luetolin, stigmasterol, kaempferol, and beta-sitosterol) were involved, targeting the proteins TOP2B (DNA topoisomerase II beta) and ADRB2 (adrenoceptor beta 2) (Supplementary Fig. 1 in Supplementary Data 10). The network data for the key herbal plants, their compounds, the proteins involved in LCTE’s effects on other COVID-19 symptoms, and information related to LCTE treatment of ‘viral respiratory infection’ are available in figures in Supplementary Data 10 and table in Supplementary Data 7.

**Fig. 4:**
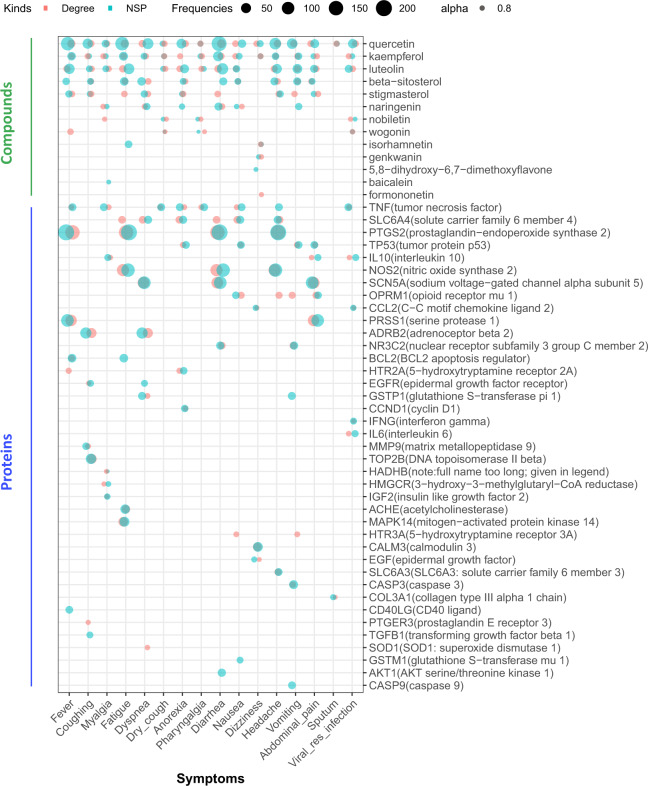
The compounds and proteins with high degrees or high occurrences in the shortest pathways. From the networks using LCTE to relieve each symptom or ‘viral respiratory infection’, the top five compounds and proteins (if there were any) were extracted based on both the vertex degree (red dots) and the frequencies with which the vertex appears in the shortest pathways (NSP) (blue dots). To avoid overlapping the dots, we used color transparency (alpha = 0.8) and dot position jitter (width = 0.2, height = 0). The dot sizes are based on the degree or the frequency at which the vertex appears in the shortest pathways. HADHB stands for hydroxyacyl-CoA dehydrogenase trifunctional multienzyme complex subunit beta, while the full names and abbreviations of other proteins are given in the plot.

In this network, the shortest pathways from the vertex of ‘LCTE’ to the vertex of one symptom (or ‘viral respiratory infection’) implies the most direct way in which LCTE affects the symptom (or ‘viral respiratory infection’). Therefore, it is reasonable to consider that the more often a vertex is located in the shortest pathways from LCTE to a specific symptom (or ‘viral respiratory infection’), the more important this vertex should be in LCTE’s symptom (or ‘viral respiratory infection’) relief. As such, compounds or proteins with high frequencies in the shortest pathways, in addition to vertices with high degrees, should be viewed as key. We summarized the top five compounds and proteins (if there were any) based on both the vertex degree and the frequency with which the vertex appears in the shortest pathways in the networks for different symptoms or ‘viral respiratory infection’ (Fig. 4), we found that the compounds quercetin, kaempferol, luteolin, beta-sitosterol, stigmasterol, and naringenin, and the proteins TNF and SLC6A4 are important to LCTE’s effects, as they are involved in the relief of more than five symptoms.

**Fig. 5:**
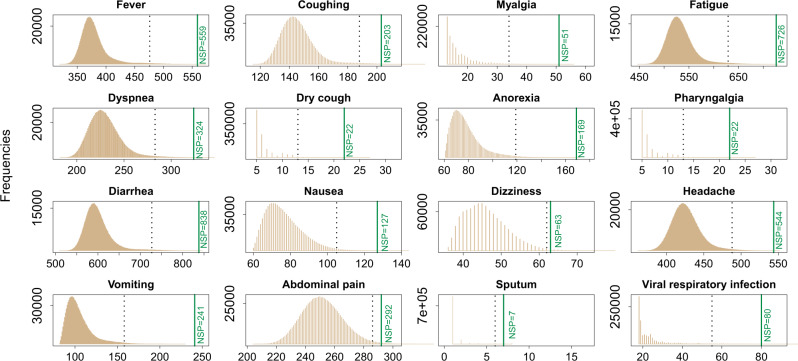
Distribution of the total number of shortest pathways for random and real LCTE networks. The brown color shows the distribution of the total number of shortest pathways from the 1,000,000 random networks. The dotted line marks the position of the 99th percentile of the distribution while the solid green line marks the value of the total number of shortest pathways from the actual LCTE network. The exact value is shown as “NSP=”.

However, the network we used for calculating a vertex’s degree or computing the frequency of a vertex’s occurrences in shortest pathways for each symptom was based on only one specific network. That is to say the network we analyzed was based on the unique combination of compounds in LCTE. In light of this, it seemed necessary to demonstrate whether or not the LCTE-specific network is superior to any random networks in relieving symptoms or ‘viral respiratory infections’. If this could be shown, then our predictions for key compounds and proteins in the LCTE network are far more likely to be genuinely important. To this end, we randomly generated compound combinations to create 1,000,000 random control networks for the real LCTE formula’s network of each symptom (or ‘viral respiratory infection’). These controls were limited to the 207 absorbable compounds found in LCTE and maintained the original formula’s compound occurrence ratio. Which compound occurred at which frequency, however, was randomly assigned (more details available in the Methods section). As shown in Fig. 5, for each symptom and ‘viral respiratory infection’, the value of the total number of shortest pathways (TNSP) from the unique, LCTE-specific network was greater than the 99th percentile of TNSP from the 1,000,000 controls. These findings demonstrate that the LCTE formula is in fact superior to random networks in terms of number of shortest pathways. It is also highly probable that, based on vertex degree or frequency of shortest pathway occurrences, the initial network pharmacologic analysis of LCTE correctly identified the compounds and proteins most important to the treatment of symptoms or ‘viral respiratory infection’.

### The general effects of LCTE soup

**Fig. 6:**
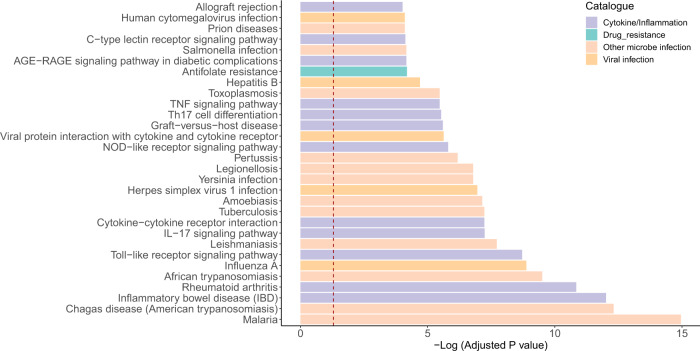
Enrichment of compound-targeted proteins in KEGG pathways. Enrichment was performed using R package *clusterprofiler* with the proteins targeted by the LCTE-contained chemical compounds as input. The bar colors correspond to different pathway functions. The vertical dot line indicated the threshold (adjusted *P* = 0.05). The exact *P* values for each pathway are given in Supplementary Data 8.

To predict the general in vivo effects of LCTE soup, we mapped all the proteins targeted by the orally-absorbable compounds to the Kyoto Encyclopedia of Genes and Genomes (KEGG) and Disease Ontology (DO) database and to find which pathways or diseases were enriched. The top 30 enriched KEGG pathways were mainly related to viral and other microbial infections, and inflammation/cytokine responses (Fig. 6). The full list of enriched KEGG pathways and the genes involved are given in Supplementary Data 8. The top two diseases enriched in DO were ‘chronic obstructive pulmonary disease’ and ‘lung disease’ (the full list of enrichment is in Supplementary Data 9). Taken as a whole, our findings suggest that that this formula’s general effects are well suited to treating viral infection in the respiratory system.

## Discussion

A fundamental requirement in prescribing any medicine to a patient is understanding the medicine’s mechanisms. With this doctrine in mind, and to provide an alternative medicine for fighting the pandemic COVID-19, we have explored the biochemical basis for LCTE’s symptom-relieving effects. Our examination into this formula’s constituents showed that LCTE contains at least two compounds, kaempferol and quercetin, that may directly inhibit the COVID-19-causing pathogen SARS-CoV-2. It also showed that most of LCTE’s main compounds are anti-inflammatory agents. Pharmacology network analysis indicated that the formula’s compounds notably target proteins related to this disease’s main symptoms, such as fever, coughing, etc., and that LCTE is generally directed toward the key disease-related pathological processes, i.e., viral infection response, inflammation/cytokine signaling, and lung diseases.

By searching three comprehensive Chinese herbal databases^7–9^ and filtering the results with ADME indices^18^ (based on the fact that Chinese herbal treatments are often boiled with water and the resulting soup then orally administered), we were able to screen out which of LCTE’s chemical components are both water-soluble and orally-absorbable. Only about 10% of the compounds passed the ADME filtration test (Table 1), indicating that when LCTE is administered as TCM, the number of bioactive compounds that enter the body is relatively small. The exclusion of such a large proportion of constituents from absorption into the body might contribute to the relative safety of traditional herbal treatments.

The usefulness of LCTE in treating COVID-19 is reasonably attributed to the pharmacological properties of the main compounds in this formula. Importantly, two ingredients have the potential to directly inhibit SARS-CoV-2. One of our recent studies screened out 13 natural compounds with the potential to directly inhibit SARS-CoV-2^19^. Two of these thirteen compounds, kaempferol and quercetin, were predicted to block SARS-CoV-2 viral replication by inhibiting 3CLpro and PLpro^19^. Kaempferol and quercetin are contained in six and seven of the plants used to formulate LCTE, resepctively (Fig. 1), meaning there is a high probability that LCTE also directly inhibits this COVID-19 cause. Moreover, of the 27 prevailing LCTE compounds, 19 are reportedly anti-inflammatory agents (Fig. 1; Supplementary Data 4). Since inflammation response is the key propagating force in COVID-19 advancement^23^, such a pharmacological profile seems well-suited to treatment.

Our protein enrichment analysis helped provide an explanation for how LCTE relieves symptoms. Proteins are key body molecules in maintaining physical functions and causing diseases, as well as principal targets for drugs. With this in mind, we identified 304 different proteins targeted by the compounds in LCTE and, based on hypergeometric distribution probability analysis, we found that many of these proteins significantly correlated to the protein sets belonging to 11 of the 15 most common COVID-19 symptoms (Fig. 2; Supplementary Data 6). These results indicate that LCTE may specifically target proteins closely related to the symptoms of this disease, especially high-occurrence symptoms such as fever, cough, and fatigue. That significant enrichment of proteins was not found for dry cough, pharyngalgia, and sputum might be due to the low number of proteins linked to these symptoms in the DisGeNET database (3, 6 and 7, respectively) (Supplementary Data 6). In addition, when DisGeNET was searched using the parameter ‘viral respiratory infection’, we found that a large number of proteins related to this disease were targeted by LCTE ingredients (Fig. 2; Supplementary Data 6). This is of note, as COVID-19 is itself a viral respiratory disease.

Through constructing and analyzing the herb-compound-protein-symptom network, we identified the key molecules involved in relieving each symptom of COVID-19. For example, the proteins PRSS1 and PTGS2, targeted by quercetin, luteolin, and stigmasterol, are important in curing fever (Fig. 3). Similar information related to other symptoms is presented in Supplementary Fig. 1 to Supplementary Fig. 15 in Supplementary Data 10 and the table of Supplementary Data 7. By combining our findings for vertices with high-degree and high shortest pathway frequency (i.e.: number of appearances on short paths between the LCTE vertex and a symptom or ‘viral respiratory infection’ vertex), we found that six compounds (quercetin, kaempferol, luteolin, beta-sitosterol, stigmasterol, and naringenin) and two proteins (TNF and SLC6A4) are important to the general effects of LCTE, as they are involved in the relief of more than five symptoms (Fig. 4). The implied significance of these compounds and proteins to LCTE’s effects is further supported by our random analysis results. In addition, the possibility that these six compounds play an important role in treating COVID-19 is consistent with reports on their anti-inflammatory properties (Fig. 1; Supplementary Data 4). That TNF targeting relates to LCTE’s symptom relief is easily understandable, as it a key pro-inflammatory cytokine; yet the roles of SCLC6A4 in LCTE’s symptom relief are not completely clear.

In order to predict the general in vivo effects, we mapped all the proteins targeted by LCTE’s compounds to the KEGG pathways and DO diseases. The results showed that the top 30 enriched KEGG pathways were related to viral and other microbial infection diseases and inflammation/cytokine signaling (Fig. 6). These pathways are closely related to pneumonia caused by coronavirus^23–25^. The top two diseases enriched in DO were ‘chronic obstructive pulmonary disease’ and ‘lung disease’, lending still more support to the assertion that LCTE is useful in treating COVID-19 (Supplementary Data 9).

Though LCTE’s effectiveness and safety have been clinically demonstrated in large numbers of Chinese patients during this COVID-19 pandemic^6^, the formula’s effectiveness and safety in treating people of other ethnicities and in different populations still requires careful evaluation. And while LCTE is rooted in TCM experience and appears to be a beneficial therapy, there are some modern medical issues that warrant further investigation. These include metabolism, compound interactions, and off-target effects of the active compounds. The influence of existing conditions, such as renal and digestive disorders, on LCTE’s application should also be addressed.

In conclusion, the clinical effectiveness of LCTE in treating COVID-19, particularly in relieving its main symptoms^6^, is rational in terms of chemical biology. LCTE contains ingredients with the potential to directly inhibit SARS-CoV-2 and inflammation, target proteins related to prevalent COVID-19 symptoms, and affect the disease’s key pathological processes.

## Methods

### Data collection

The chemical compounds of each of the twenty plants used to make LCTE were found by examining three comprehensive Chinese herbal databases: the Traditional Chinese Medicine Systems Pharmacology database (TCMSP, http://www.tcmspw.com/)^7^, the Encyclopedia of Traditional Chinese Medicine (ETCM, http://www.nrc.ac.cn:9090/ETCM/)^9^ and SymMap (https://www.symmap.org/)^8^. Since Chinese herbal treatments are prepared by boiling herbs in water and the collected soup is taken orally, an in silico integrated model of absorption, distribution, metabolism, and excretion (ADME) was used to screen the bioactive compounds of orally-administered LCTE. The indices used for the screening included oral bioavailability evaluation, Caco-2 permeability, drug-like value, and drug half-life. The threshold values indicating effectiveness for these four indices were >30%, >–0.4, >0.18, and >3 h, respectively as recommended^18^. The values of these four indices can be obtained from the TCMSP database. Each compound passing ADME screening was cross-checked in the three aforementioned Chinese herbal databases to find the compound’s protein targets.

The proteins related to the main symptoms of COVID-19 were found through searching the DisGeNET (https://www.disgenet.org/)^22^ database using the name of each symptom as input. Since COVID-19 is a viral respiratory infection, we also searched the DisGeNET database using the input ‘viral respiratory infection’ to find the related proteins.

Literature about the anti-inflammation properties of the main compounds was retrieved by searching PubMed with each chemical compound’s name and ‘inflammation’ as query terms.

### Protein enrichment

Enrichment of the COVID-19 symptom-related proteins targeted by LCTE ingredients were assessed by the probability (*P*) value of hypergeometric distribution:

$$P(x \ge k) = {\mathrm{C}}_k^M{\mathrm{C}}_{n - k}^{N - M}{\mathrm{/C}}_n^N/,{\mathrm{with}}\,{\mathrm{C}}_n^N = N!/n!\left( {N - n} \right)!$$

*k* represents the number of overlapping proteins between the protein set targeted by LCTE-contained compounds and the set of protein related to each symptom or ‘viral respiratory infection’. *M* represents the number of proteins in the set related to a single symptom or ‘viral respiratory infection’, *n* stands for the number of proteins in the set targeted by LCTE ingredients. *N* (the total number of proteins) was set as 17,549, which is the number of genes included in the latest version of DisGeNET RDF (v6.0). The adjusted *P* value was obtained with the *p.adjust* function of R package *stats*.

### Network analysis

The constructions, degree calculations, and frequencies of vertices in the shortest pathways of the plants-compound-protein-symptom network were performed using R package *igraph* (v1.2.4.2) and the results are presented with Cytoscape (v3.7.2). The network constructions are based on using these data as edges: herbal plants and their corresponding chemical compounds, chemical compounds and their affected proteins, and proteins related to each symptom. For specific symptoms or ‘viral respiratory infection’, the network was constructed independently. When constructing a network for a specific symptom or ‘viral respiratory infection’, the proteins used were different in that only the proteins related to the symptom or ‘viral respiratory infection’ were included in the construction. The shortest pathways are the ones with the least vertices between the vertex of “LCTE” and the vertex of the symptom or ‘viral respiratory infection’. This information was extracted by the function ‘*get.all.shortest.paths*’ of R package *igraph*. After extracting the shortest pathways, the frequency of each vertex in the shortest pathways was calculated.

To evaluate the superiority of LCTE in treatment, in this study, for each symptom and for ‘viral respiratory infection’, we compared the total number of shortest pathways in the actual LCTE network to 1,000,000 random networks similar to the original. LCTE has one unique combination of compounds in that each distinct compound appears a certain number of times in the formula; i.e.: 1 of these compounds appears eight times, 1 appears seven times, 3 appear six times, 2 appear four times, 3 appear three times, 17 appear twice, and a majority, 180 compounds, are present only once. Our control networks were constructed based on random combinations of these compounds. Each random combination of compounds was made by selecting only from the 207 compounds found in the real LCTE formula. The frequency ratios remained the same as well, but we randomly assigned which compound occurred at which frequency. This process was accomplished via the sampling without replacement method in which we randomly drew 1, 1, 3, 2, 3, and 17 compounds and assigned them with frequencies of 8, 7, 6, 4, 3, and 2 times, respectively. The remaining 180 compounds were set to a frequency of one (as per above). Based on the features of the network and to reduce the workload, we used the following equation to find the total number of shortest pathways from the random formula’s vertex to the vertex of one symptom or ‘viral respiratory infection’ in each network:

$${\mathrm{the}}\ 	{\mathrm{total}}\ {\mathrm{number}}\ {\mathrm{of}}\ {\mathrm{shortest}}\ {\mathrm{pathway}}\\ 	= \Sigma _{{\mathrm{i = 1}}}^{207}\, ({\mathrm{frequencies}}\ {\mathrm{randomly}}\ {\mathrm{assigned}}\ {\mathrm{to}}\ {\mathrm{the}}\ i{\mathrm{th}}\ {\mathrm{compound}}\times {\mathrm{the}}\ {\mathrm{number}} \\ 	{\mathrm{of}}\ {\mathrm{shortest}}\ {\mathrm{pathway}}\ {\mathrm{from}}\ {\mathrm{the}}\ {\mathrm{vertex}}\ {\mathrm{of}}\ i{\mathrm{th}}\ {\mathrm{compound}}\ {\mathrm{to}}\ {\mathrm{the}}\ {\mathrm{vertex}}\ {\mathrm{of}}\\ 	{\mathrm{symptom}}\ {\mathrm{or}}\ {\hbox{`}}{\mathrm{viral}}\ {\mathrm{respiratory}}\ {\mathrm{infection}}{\hbox{'}}\ {\mathrm{in}}\ {\mathrm{the}}\ {\mathrm{real}}\ {\mathrm{LCTE}}\ {\mathrm{network}})$$

In the above equation, the number of shortest pathways from the vertex of *i*th compound to one symptom (or ‘viral respiratory infection’) for the actual LCTE network was obtained with R package igraph software. After finding the total number of the shortest pathways for each random network, the distribution percentile for the total number of shortest pathways in the 1,000,000 random networks was calculated.

### General in vivo effects prediction

The general effects of LCTE were predicted by KEGG pathway and Disease Ontology (DO) enrichment. The full list of proteins targeted by the LCTE-containing compounds is mapped to these two databases, and significantly enriched results (with the adjusted *P* value cutoff set to 0.05) were extracted. The analyses were carried out by the R package *clusterProfiler* (v3.14.2) functions *enrichKEGG* and *enrichDO* and the dependent R package was installed and activated in advance as instructed.

### Statistics and reproducibility

All data were processed using statistical language R (v3.6.2) unless otherwise specified. Protein enrichment, KEGG pathway enrichment, and DO disease enrichment have been detailed above. The shortest pathways in the real LCTE formula network were extracted by package *igraph*, and the shortest pathways for the random control networks were calculated using the equation given above. The percentiles of the total number of shortest pathways for the random controls were calculated with *quantile* function of R package *stats* (v3.6.2). Adjusted *P* < 0.05 was considered to be of statistical significance. Plots were produced with R *gglopt2* (v3.3.2) software and Cytoscape (v3.7.2).

### Reporting summary

Further information on research design is available in the Nature Research Reporting Summary linked to this article.

## Supplementary information

Supplementary Data 1

Supplementary Data 2

Supplementary Data 3

Supplementary Data 4

Supplementary Data 5

Supplementary Data 6

Supplementary Data 7

Supplementary Data 8

Supplementary Data 9

Supplementary Information

Description of Additional Supplementary Files

Reporting Summary

## Data Availability

The authors declare that all data supporting the findings of this study are available within the manuscript and its supplementary files. There are ten supplementary files, including two PDF format files (Supplementary Information, including Supplementary Figs. 1–15; and Supplementary Data 1, for preparing LCTE soup) and eight Microsoft Excel format files (Supplementary Data 2–9). Supplementary files are also available at Figshare^26^.
