# Supplementary material for: Network pharmacology suggests biochemical rationale for treating COVID-19 symptoms with a Traditional Chinese Medicine
Source: Commun Biol. 2020 Aug 18;3:466. doi: 10.1038/s42003-020-01190-y (PMC7434773; doi:10.1038/s42003-020-01190-y)
Supplement: Supplementary file 1 — Supplementary Data 1 [file 42003_2020_1190_MOESM1_ESM.pdf]

The protocol for preparing LCTE medicine

1. Buy the materials and weight out each component (detailed in Table 1), the weight should be two times the dose in the Table 1 for oral administration *b.i.d*.
2. Washing all the materials for three times with cold water.
3. Soak the 20 herbal plants in cold water for 45 min.
4. Put the *rudis gypsi miscueris* in pot, add with water to cover it, and boil for 45 min.
5. Take out the soaked herbal plants and added to the pot that boiling the *rudis gypsi miscueris*.
6. Add water to the pot with the water surface to cover all the plants.
7. Boil the plants and *rudis gypsi miscueris* till the water remained in the pot is about 200 ml.
8. Collect the soap in the pot.
9. Add cold water again to the pot with plants and *rudis gypsi miscueris* and repeated the step 7 and 8.
10. Mix the soap collected two times together, and put the mixed soap *i.e.*, the medicine in refrigerator.
11. The medicine soap are orally administrated two times a day, at 40 minutes after breakfast and dinner, respectively, each time with 200 ml. the Soap should be heat to warm before intake.
12. One therapeutic regimen consists three continuous days. The patients could be prescribed for 2 regimens.

Note: the herbal medicine could be prepared for several patients at same time by scaling up the components amounts with the number of the patients.
