# Supplementary material for: Network pharmacology suggests biochemical rationale for treating COVID-19 symptoms with a Traditional Chinese Medicine
Source: Commun Biol. 2020 Aug 18;3:466. doi: 10.1038/s42003-020-01190-y (PMC7434773; doi:10.1038/s42003-020-01190-y)
Supplement: Supplementary file 10 — Supplementary Information [file 42003_2020_1190_MOESM10_ESM.pdf]

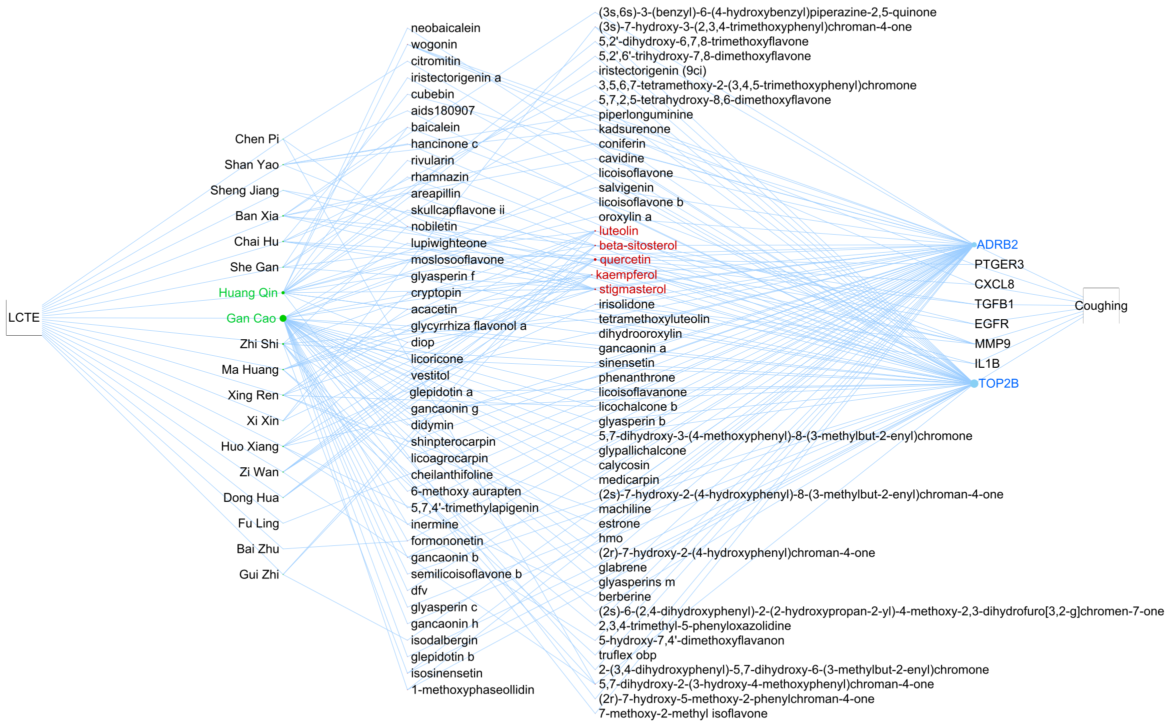

Supplementary Figure 1. The network of plants, chemical compounds and target proteins for relieving coughing by LCTE

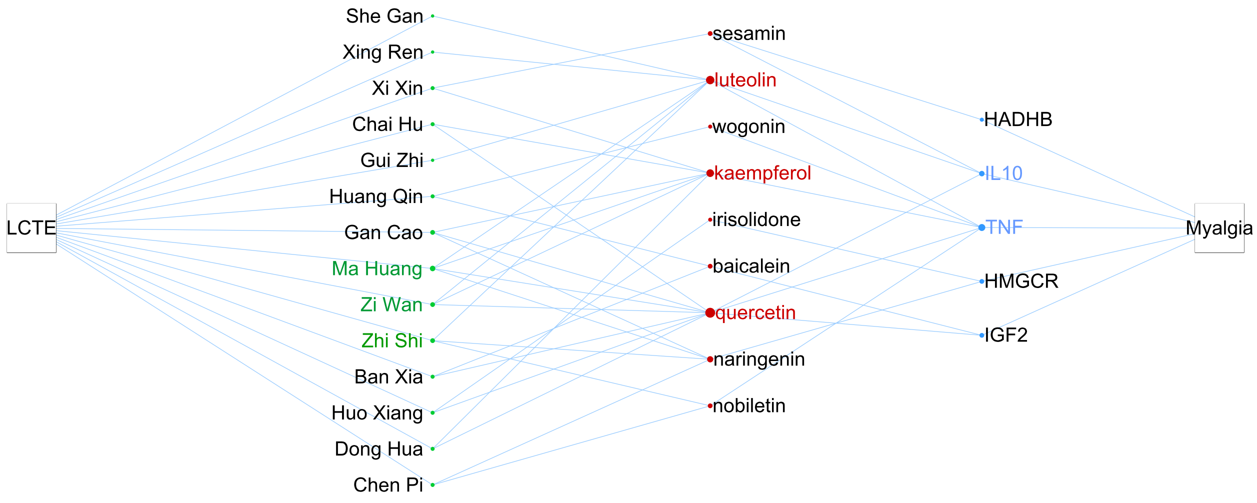

Supplementary Figure 2. The network of plants, chemical compounds and target proteins for relieving myalgia by LCTE

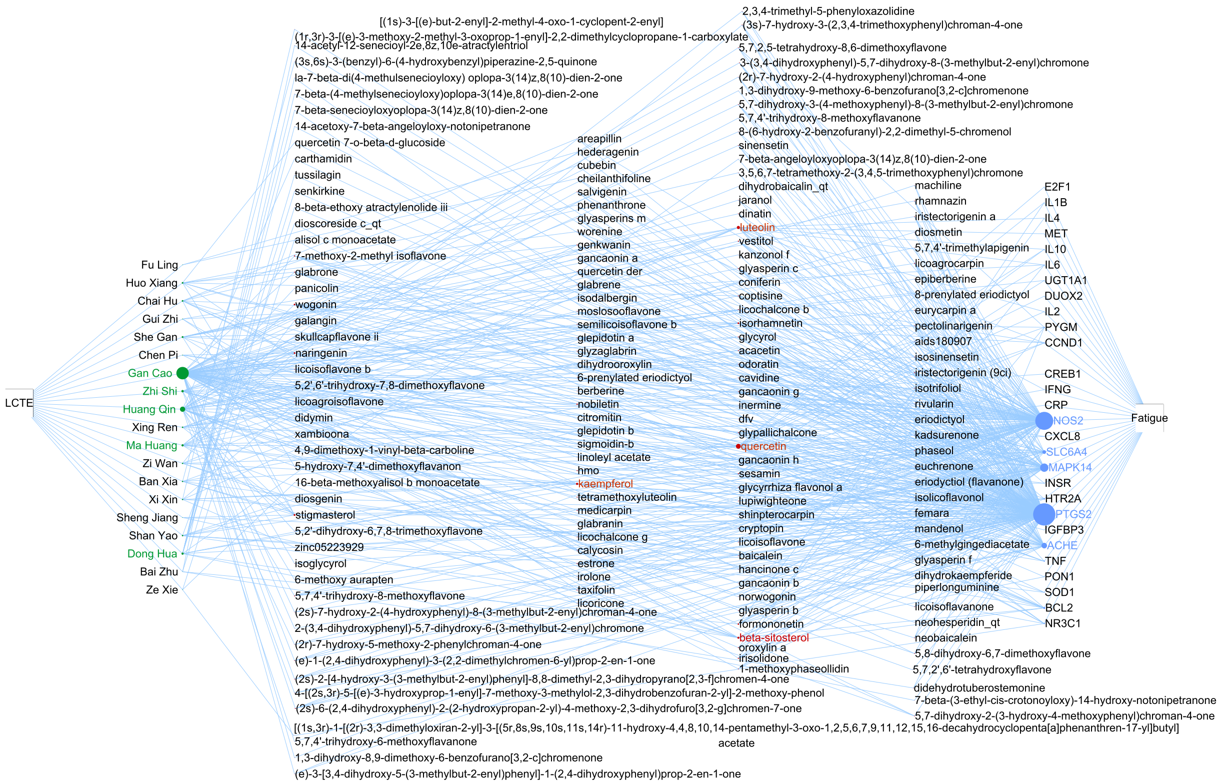

Supplementary Figure 3. The network of plants, chemical compounds and target proteins for relieving fatigue by LCTE

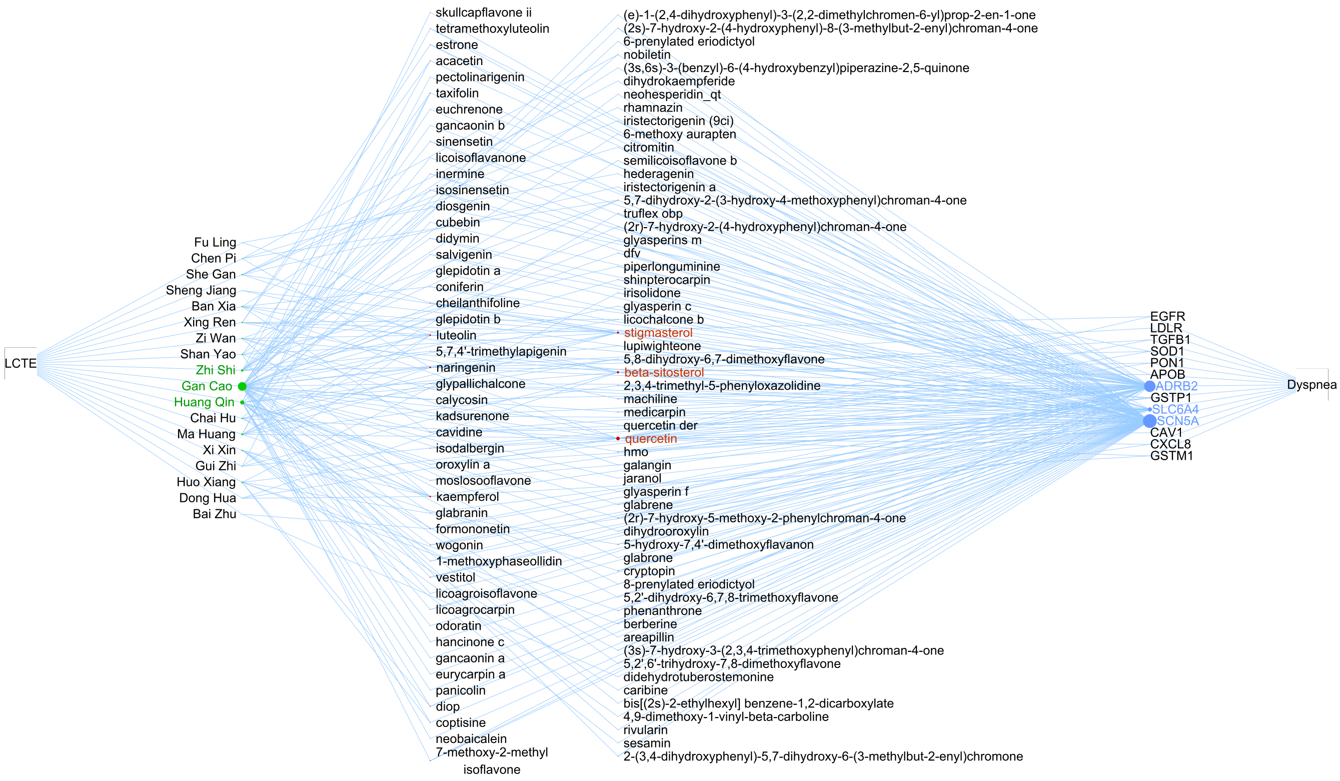

Supplementary Figure 4. The network of plants, chemical compounds and target proteins for relieving dyspnea by LCTE

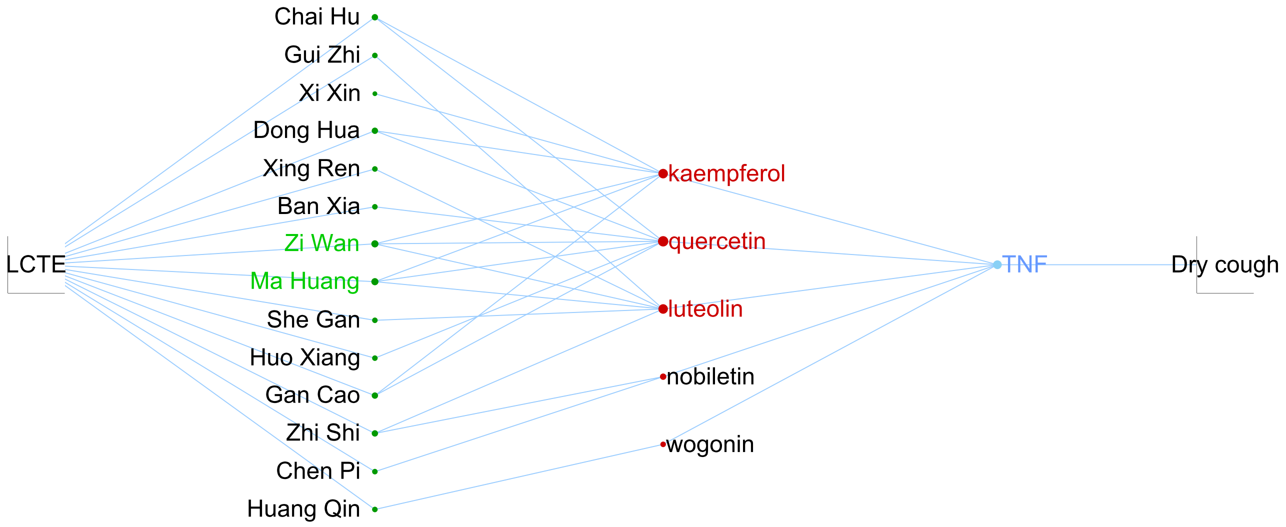

Supplementary Figure 5. The network of plants, chemical compounds and target proteins for relieving dry cough by LCTE

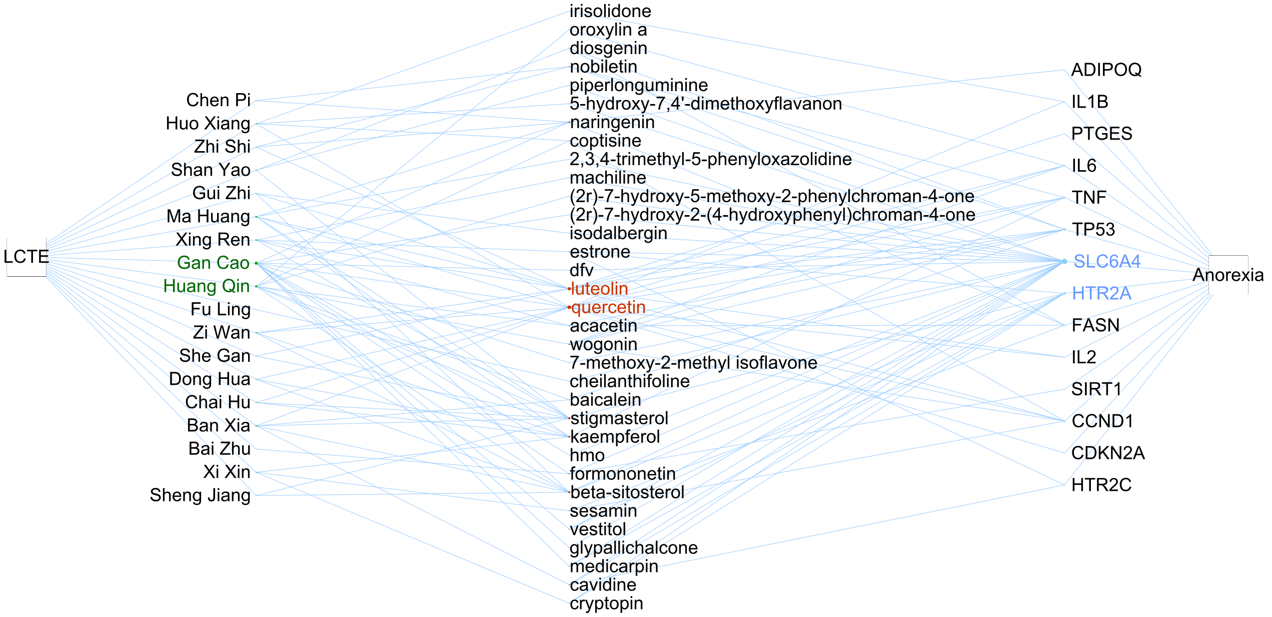

Supplementary Figure 6. The network of plants, chemical compounds and target proteins for relieving anorexia by LCTE

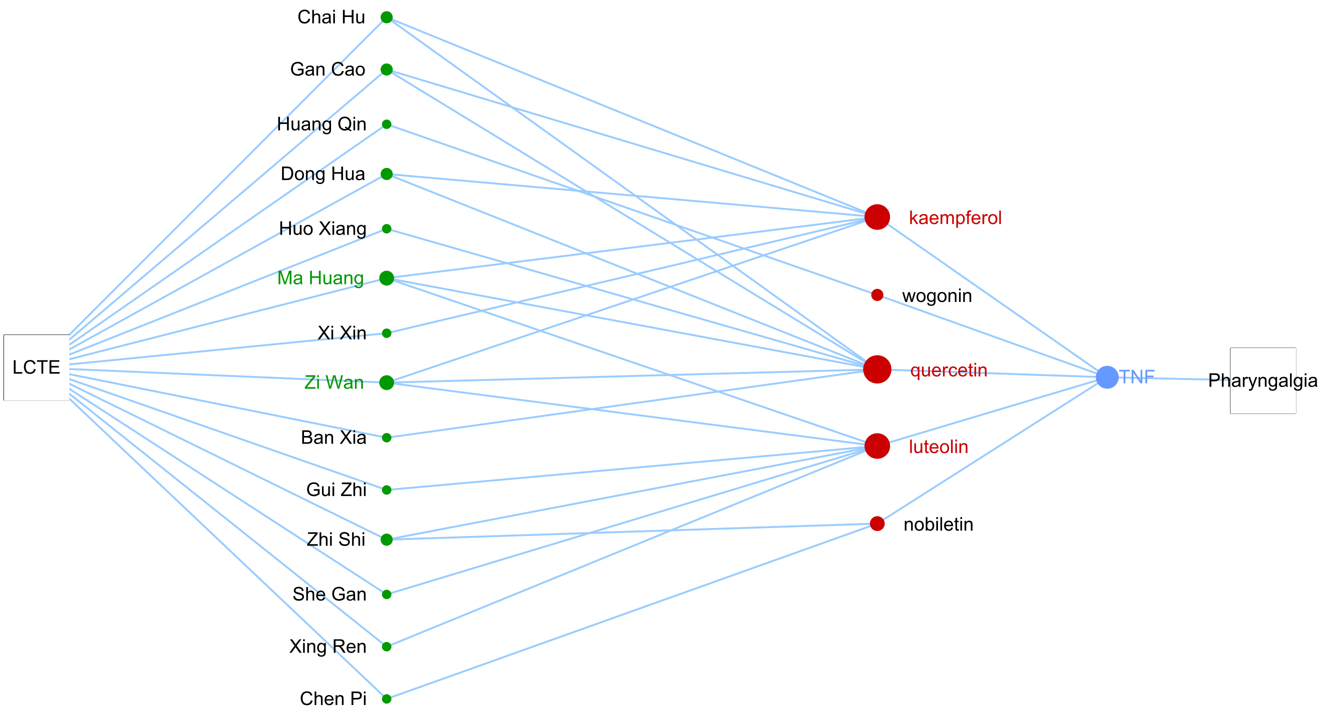

Supplementary Figure 7. The network of plants, chemical compounds and target proteins for relieving pharyngalgia by LCTE

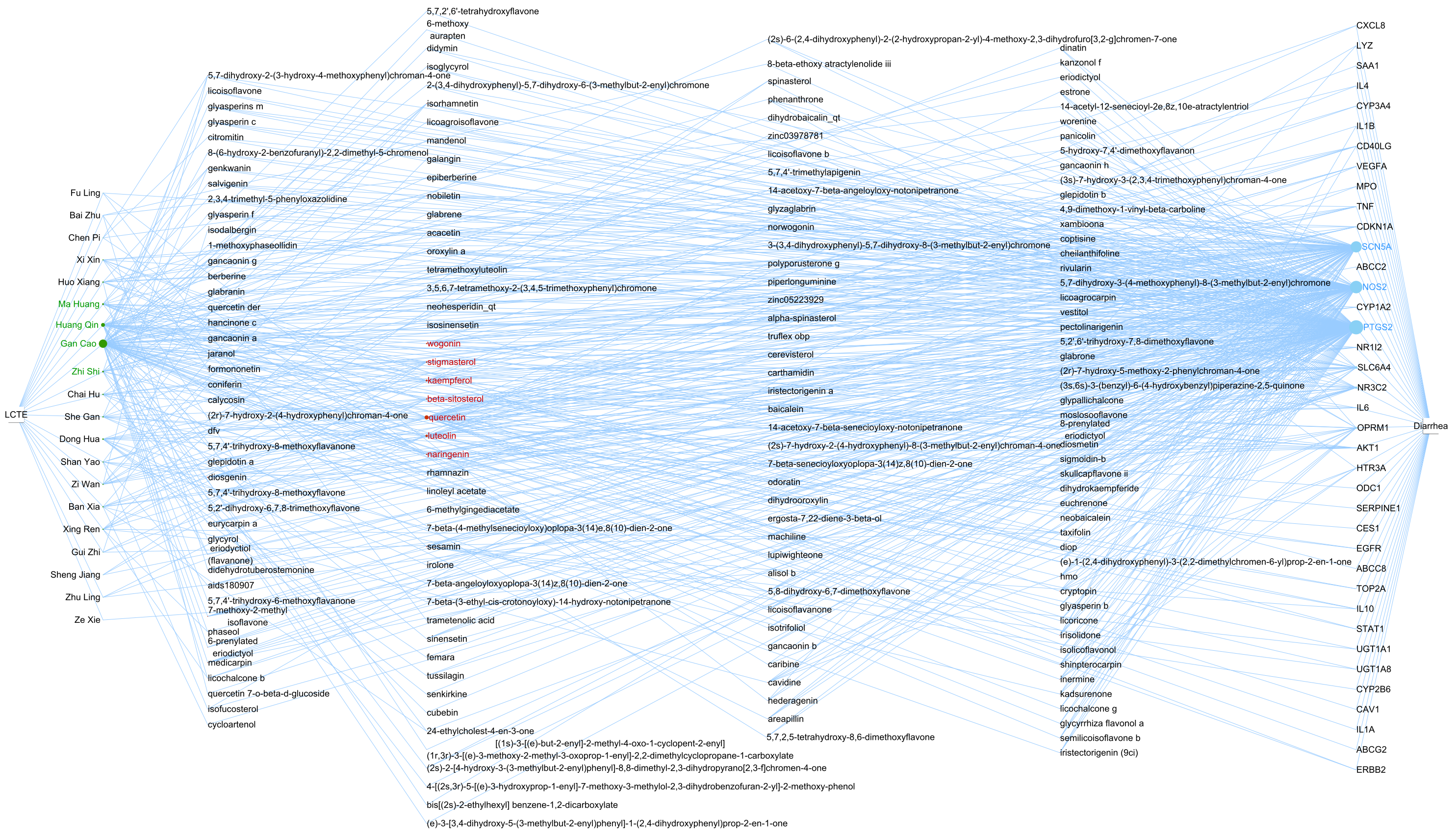

Supplementary Figure 8. The network of plants, chemical compounds and target proteins for relieving diarrhea by LCTE

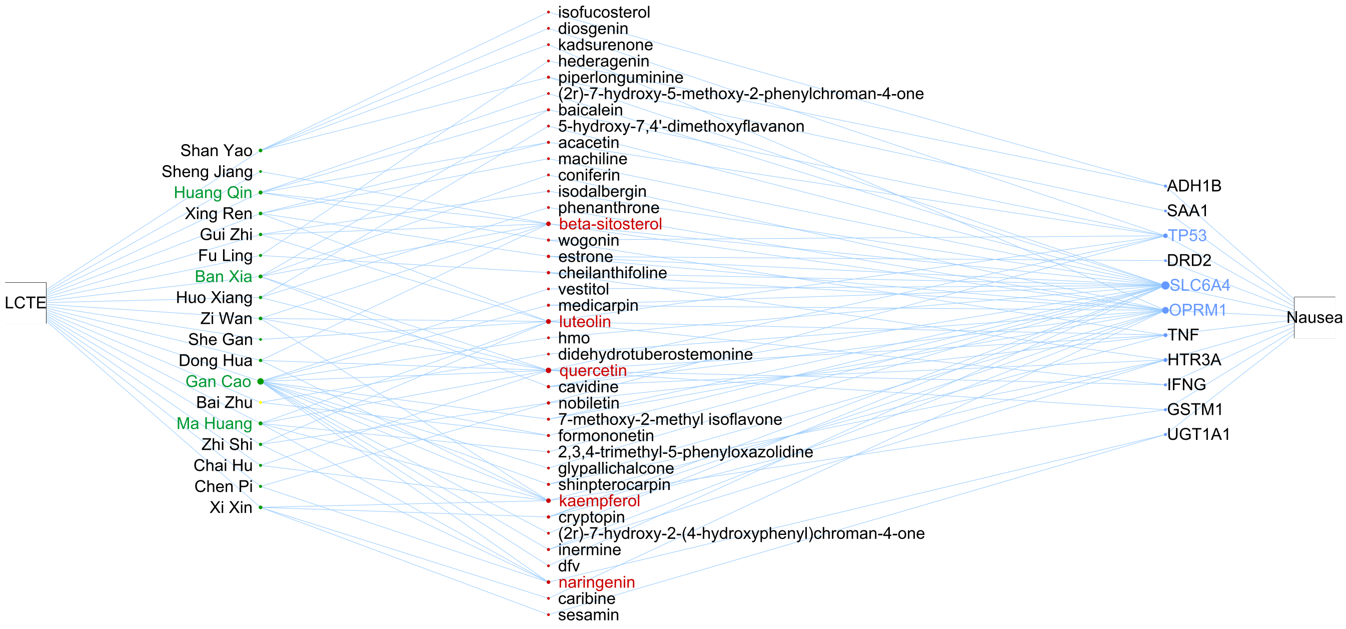

Supplementary Figure 9. The network of plants, chemical compounds and target proteins for relieving nausea by LCTE

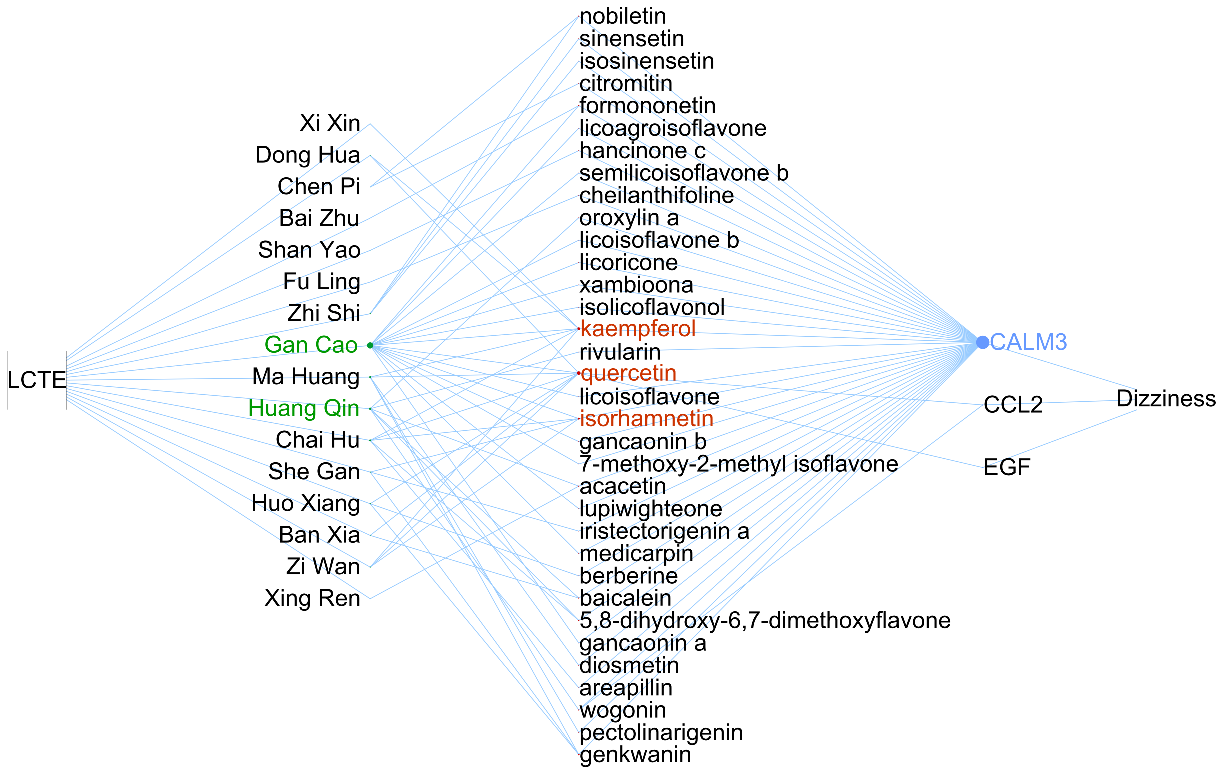

Supplementary Figure 10. The network of plants, chemical compounds and target proteins for relieving dizziness by LCTE

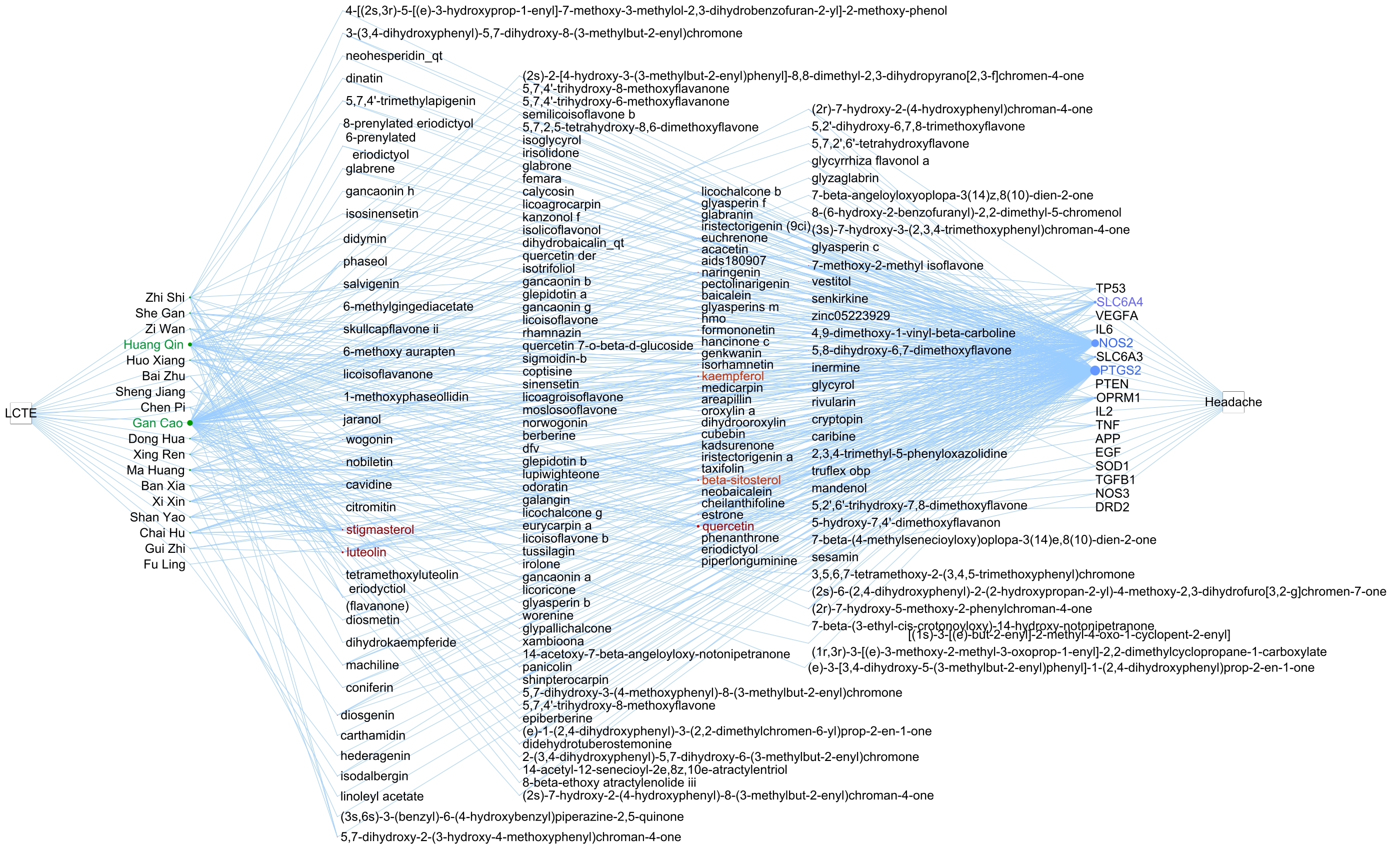

Supplementary Figure 11. The network of plants, chemical compounds and target proteins for relieving headache by LCTE

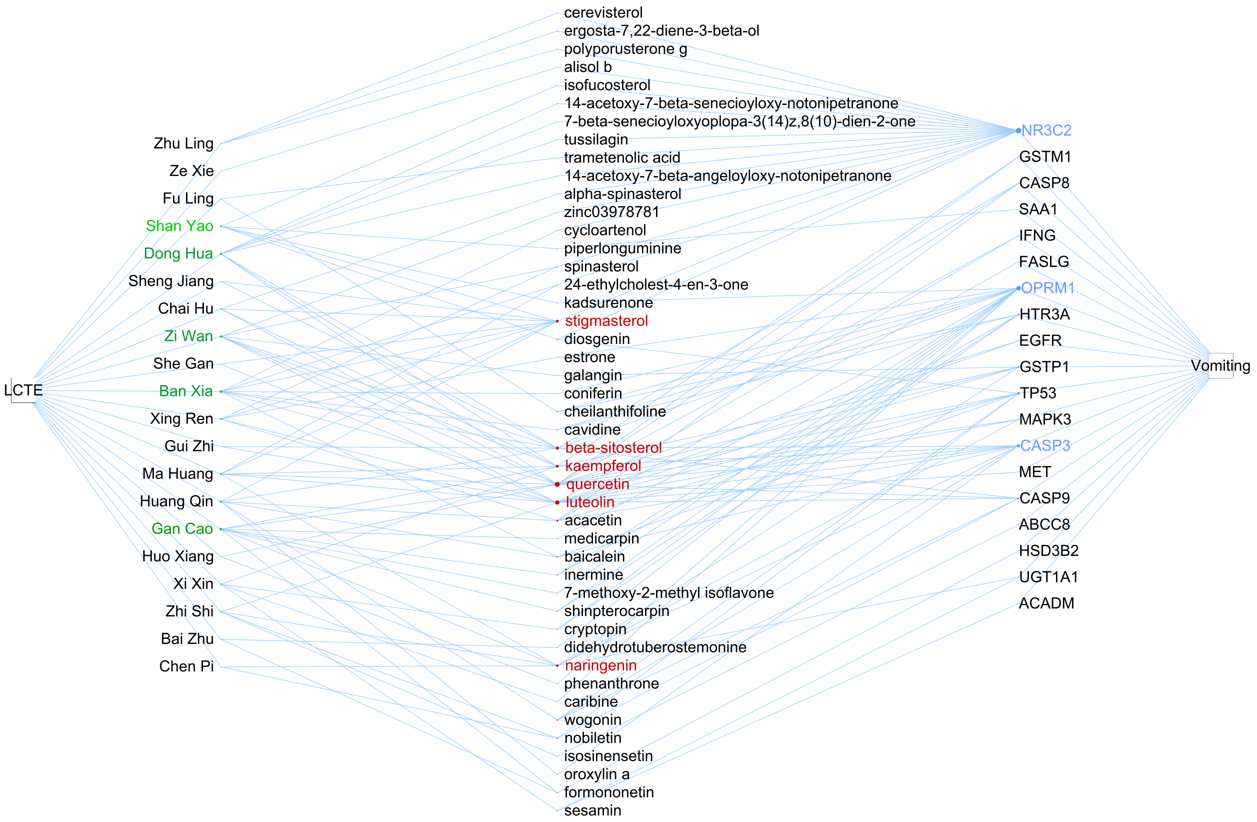

Supplementary Figure 12. The network of plants, chemical compounds and target proteins for relieving vomiting by LCTE

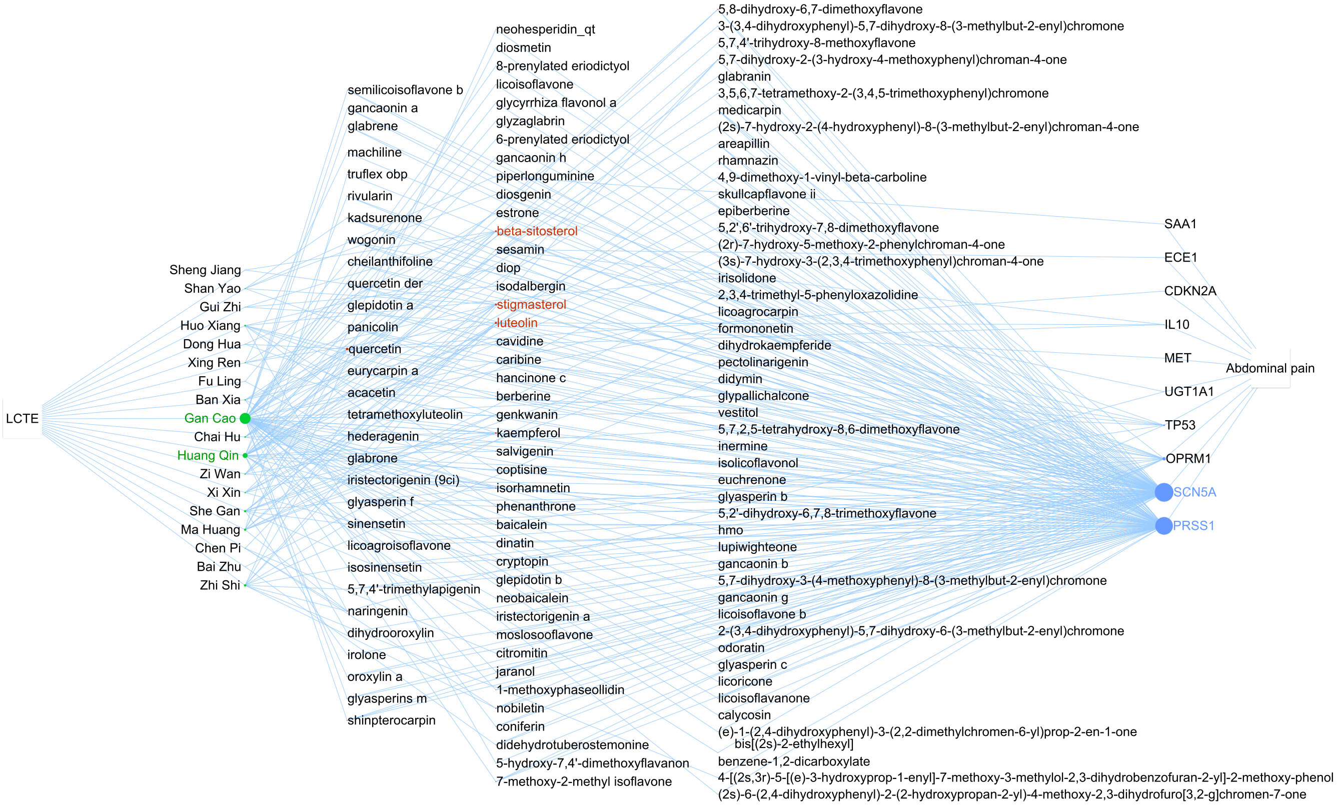

Supplementary Figure 13. The network of plants, chemical compounds and target proteins for relieving abdominal pain by LCTE

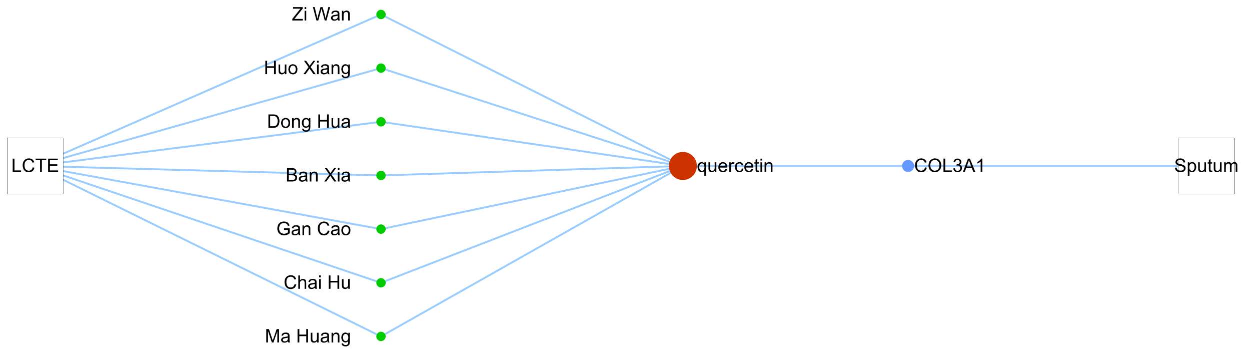

Supplementary Figure 14. The network of plants, chemical compounds and target proteins for relieving sputum by LCTE

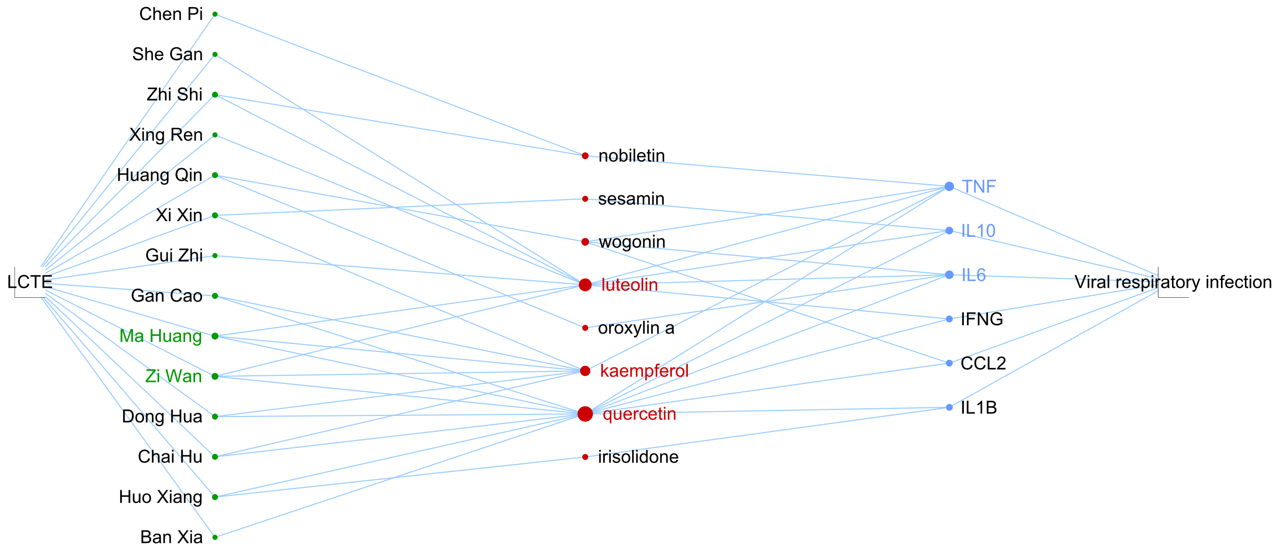

Supplementary Figure 15. The network of plants, chemical compounds and target proteins for treating viral respiratory infection by LCTE
