# Supplementary material for: Network pharmacology suggests biochemical rationale for treating COVID-19 symptoms with a Traditional Chinese Medicine
Source: Commun Biol. 2020 Aug 18;3:466. doi: 10.1038/s42003-020-01190-y (PMC7434773; doi:10.1038/s42003-020-01190-y)
Supplement: Supplementary file 11 — Description of Additional Supplementary Files [file 42003_2020_1190_MOESM11_ESM.pdf]

Description of Additional Supplementary Files

File Name: **Supplementary Data 1**
Description The ways for preparing the LCTE soup

File Name: **Supplementary Data 2**
Description Chemical compounds contained in each LCTE's plant

File Name: **Supplementary Data 3**
Description The protein targets of each chemical ingredient in LCTE

File Name: **Supplementary Data 4**
Description The occurrences of the chemical ingredients in LCTE and the anti-inflammatory reports for the main ingredients

File Name: **Supplementary Data 5**
Description The proteins related to main symptoms of COVID-19 and the disease of viral respiratory infection

File Name: **Supplementary Data 6**
Description The enrichment and proteins overlapped between ingredients-targeted and symptoms or disease-related proteins

File Name: **Supplementary Data 7**
Description Summary of important LCTE plants, chemical compounds, and protein targets for relieving each COVID-19 symptom and treating viral respiratory infection

File Name: **Supplementary Data 8**
Description Full list of KEGG pathways enriched for proteins targeted by LCTE chemical compounds

File Name: **Supplementary Data 9**
Description Full list of Disease Ontology enriched for proteins targeted by LCTE chemical compound
